# 

**Published:** 1888-04-14

**Authors:** 


					UIML HL.NN Y.T^T W W ? f
P  \ \  Ml ml litij Per Annum, 6s. 6d.. post free.
No. 81, VoL IV.-
-April 14th, 1888.
nr w irv*
Monthly Parts, 6d.; post free, 7id.
I Registered at the General Post Office WL ^ \J   '
ns a iVewspnper.] Ditto per Ann., 7s. ed. post free.

				

